# Deep Learning Approach for Ascaris lumbricoides Parasite Egg Classification

**DOI:** 10.1155/2021/6648038

**Published:** 2021-04-26

**Authors:** Narut Butploy, Wanida Kanarkard, Pewpan Maleewong Intapan

**Affiliations:** ^1^Dept. of Computer Engineering, Khon Kaen University, Khon Kaen 40002, Thailand; ^2^Dept. of Parasitology, Khon Kaen University, Khon Kaen 40002, Thailand

## Abstract

*A. lumbricoides* infection affects up to 1/3 of the world population (approximately 1.4 billion people worldwide). It has been estimated that 1.5 billion cases of infection globally and 65,000 deaths occur due to *A. lumbricoides*. Generally, allied health classifies parasite egg type by using on microscopy-based methods that are laborious, are limited by low sensitivity, and require high expertise. However, misclassification may occur due to their heterogeneous experience. For their reason, computer technology is considered to aid humans. With the benefit of speed and ability of computer technology, image recognition is adopted to recognize images much more quickly and precisely than human beings. This research proposes deep learning for *A. lumbricoides*'s egg image recognition to be used as a prototype tool for parasite egg detection in medical diagnosis. The challenge is to recognize 3 types of eggs of *A. lumbricoides* with the optimal architecture of deep learning. The results showed that the classification accuracy of the parasite eggs is up to 93.33%. This great effectiveness of the proposed model could help reduce the time-consuming image classification of parasite egg.

## 1. Introduction

Intestinal parasites are among the main public health problems around the world especially in tropical and subtropical countries [1]. Ascaris lumbricoides is a nematode parasite that causes the common tropical infection ascariasis in humans [2]. This parasite causes harmfully infection in human digestive tract. The studies have shown that the parasite survive for 1 to 2 years in human body [3]. The female worms produce about 200,000 eggs. There are three forms of eggs: fertile, decorticate, and infertile. Fertile eggs are oval in shape, measuring 40×60 *μ*m. The egg is termed decorticate if the external albuminous layer is absent. Infertile eggs are larger, measuring 60×90 *μ*m and more elongated in shape, have a thinner shell, and are poorly organized internally, being a mass of variably sized granules. Nowadays, advance in machine learning is able to recognize and classify images precisely, which can be used to assist doctors in diagnosing parasitic imaging. Nkamgang et al. [4] detect and automatically detect intestinal parasites by neuro-fuzzy system. Research by Poostchi et al. [5] explores the use of machine learning that can improve performance in the field of human parasite physician. In addition to the aforementioned machine learning technology, there is also a technology called convolutional neural network (CNN) deep learning, which is the most effective and popular for visual recognition in present [6, 7]. An example of clinical application using CNNs is Zhicheng et al.' study [8] which offers deep learning for the classification of breast cancer images, which give more efficient image recognition. Zou et al. [9] adopt CNN deep learning to classify mammographic breast cancer diagnosis. Tiwari et al.'s research [10] applied CNNs for classification then compared against Naïve Bayes (NB) and Support Vector Machine (SVM). The results showed that CNNs are more accurate than both NB and Support Vector Machine (SVM). From the research works, CNN deep learning is used to improve the recognition and image classification accuracy of three A. lumbricoides types. The goal is to create a reliable model that can help clinicians accurately and quickly visualize parasite.

## 2. Materials and Methods

### 2.1. Convolution Neural Networks

Convolution Neural Network (CNN) deep learning is an extraction of multilayered visual features, to build a neural network for increasing the traditional neural network capability [7, 11]. CNN learning architecture creates complex learning process because of the large number of extracted features. Thus, the processing must be performed parallel. Supporting resources must be shared between the central processing units (CPUs) and the graphics processing units (GPUs). Extraction of the featured images will be adjusted with the simultaneously. Therefore, after each round of learning, the characteristic filters will be adjusted to suit the job. Each attribute extraction generates a feature map containing fields connected to the neuron in the previous layer. Sometimes, the pooling layer is inserted between the convolution layers to reduce the spatial size, reducing the number of parameters and calculations in the network. In addition, the learning weight must be adjusted with an activation function.

**Figure 1:**
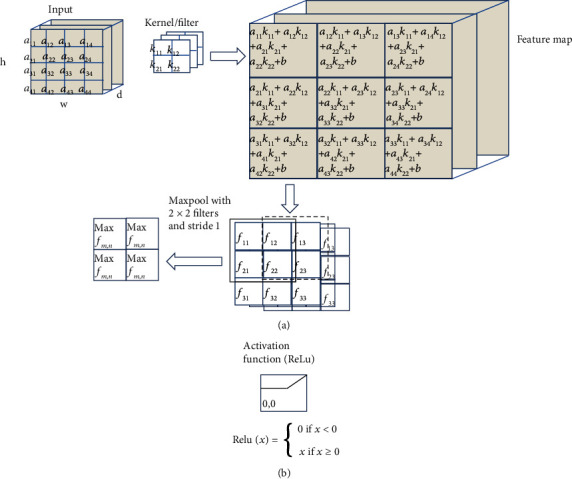
Creating the (a) convolution layer is the function of the convolution layer and pooling, which contains the import image and filter feature map obtained by finding the relationship between the images imported to the kernel; then, reducing the size by pooling using a (b) 2 × 2 filter is an example of the performance of the ReLu activation function. This function is inserted in the convolution process to send the learning value back as far as possible.

Figure 1(a) shows an example of a convolution layer, starting with a 4 × 4 import image to calculate the features of the image with a 2 × 2 filter. The calculations can be performed according to the equation (1).

(1) Ck=mnWk∗Fmn+bk,

where *k* is the location of the neuron, *F* is the filter for the feature map, *W*_*k*_ is the image location to be extracted, the dominant feature *b* is the bias, and (*C*_*k*_)_*mn*_ is the extraction result. Once a feature map is created, want to simplify the computation can add a functional layer, called maxpooling, which selects the agent that provides maximum information, as in the example equation. For example, filters are Sobel filters: generally used to highlight edges; Gaussian filters: generally used to remove noise; Emboss filters: generally used to accentuate brightness differences.  Figure 1(b) shows an example of the calculation of the rectified linear activation function (ReLu).

### 2.2. Artificial Neural Network

Artificial neural network (ANN) is derived from a biological network of neurons [12, 13]. In the ANN model, a collection of nodes termed as neurons constitute a layer that can be used for different tasks, such as prediction, classification, and pattern recognition. One of the main advantages of ANN is the opportunity to retrieve hidden information that allows solving complex problems [5]. ANN has three main layers that are interconnected. The first layer consists of input neurons. Those neurons send data onto the second layer are called the hidden layer, which in turn sends the output neurons to the third layer. The input units receive various forms and structures of information based on an internal weighting system, and the neural network attempts to learn about the information presented to produce one output.

### 2.3. CNN Deep Learning Architecture

CNN deep learning shares two main functions: extraction with convolution (Figure 1) and neural network learning. It is a neural network with several hidden layers. The basic architecture of CNNs consists of layers. Convolutional and maxpooling [14] finally build a neural network for image recognition with a fully connected layer.

**Figure 2:**
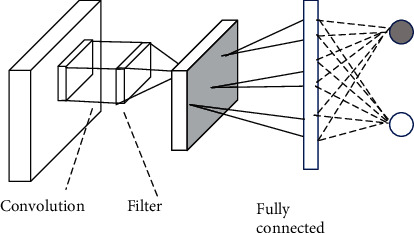
A typical convolutional neural network architecture.

CNN is a multilayer perceptron neural network which is thought to allow computers to learn many steps in a parallel manner.  Figure 2 consists of a feature map layer, and each feature map represents a particular feature extracted at the locations of the associated input [15]. The more learning, the deeper the computer will be able to predict the incoming data more accurately. The last layer is fully connected layer that is connected to all neurons in the previous layer. It can be denoted as equation (2).

(2) $$\begin{array}{c} y_{k}=\left(\underset{p}{\sum }W_{kp}x_{p}+b_{k}\right), \end{array}$$

where *x*_*p*_ is the *p* input neuron, *y*_*k*_ is the *k* output neuron, *W*_*kp*_ denotes the weight connecting *x*_*p*_ with *y*_*k*_, and *b*_*k*_ denotes the bias term of *y*_*k*_.

In general, the CNN connection architecture is divided into two layers: the extract feature (convolution layer) and the learn with the neural network (fully connected layer), but to extract the feature, besides the filter, the function must be added to achieve the feature and learning. The speed of images using the activate function, downsampling with maxpooling, and reducing the number of nodes with dropout can be described as follows.

*Overfitting*: the problem with overfitting a model is that it is a scam, because it may measure the accuracy of the learning model, and it is very effective, but when using a model for predictive unseen data, it predicts that it is less accurate.

*Rectified linear unit (ReLu)*: this activation function and its variants show superior performance in many cases and are the most popular activation function in deep learning [16]. Therefore, it trains several times faster than their equivalents with other activation functions.

*Maxpooling*: the maxpooling also downsampling for the spatial dimension of the input [15, 17] maxpooling is a pooling operation that selects the maximum element from the region of the feature map covered by the filter. Thus, the output after the maxpooling layer would be a feature map containing the most prominent features of the previous feature map.

*Dropout*: the dropout is a regularization method that stochastically sets to zero the activations of hidden units for each training case at training time it prevents overfitting [18]. The neurons which are dropped in this way do not contribute to the forward pass and do not participate in back-propagation [19].

### 2.4. Dataset

**Figure 3:**
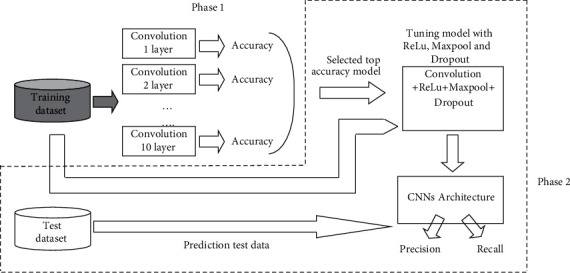
The proposed deep learning for parasite recognition.

The datasets are freshly prepared for the *A. lumbricoides*-infected stool samples by using a gold standard formalin ethyl acetate concentration technique [20]. The datasets in our methodology consist of two phases as described by Figure 3 and using *A. lumbricoides* eggs from the Department of Parasitology, Faculty of Medicine Khon Kaen University, Thailand. The dataset is separate into training and testing set, and both training and testing set consist of three *A. lumbricoides* eggs, namely, (1) infertile egg, (2) fertile egg, and (3) decorticate egg.

**Figure 4:**
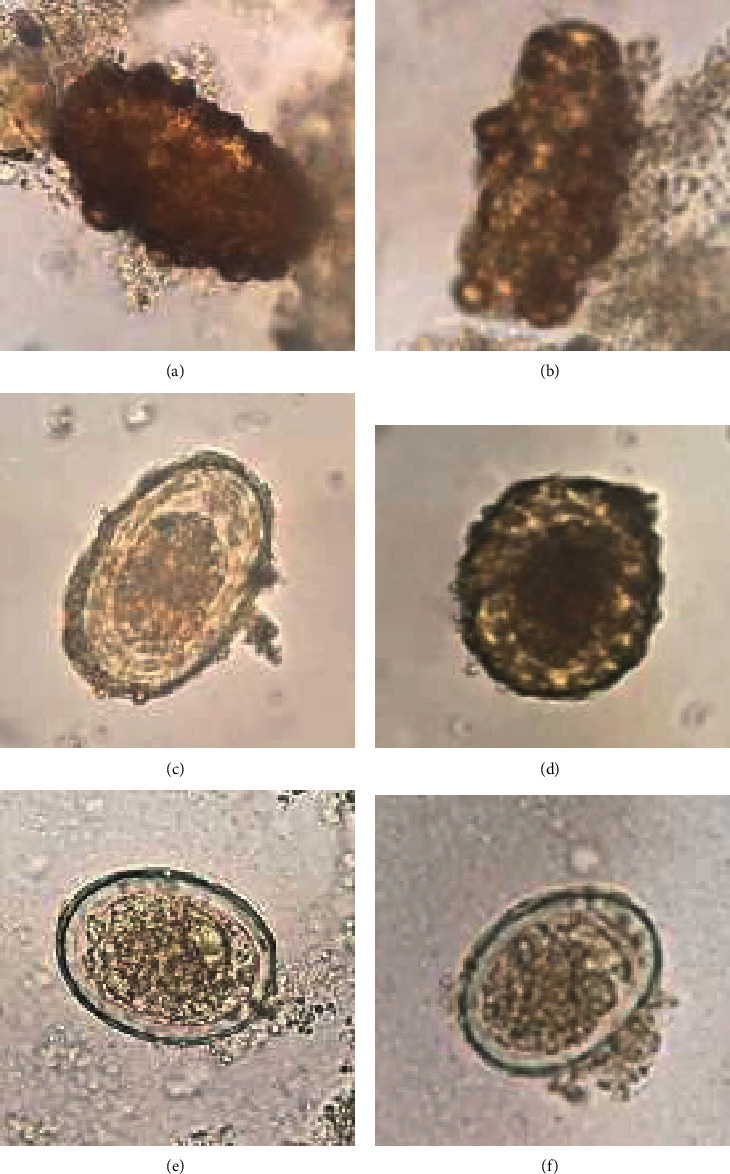
The three types of *A. lumbricoides* eggs.

Figure 4 shows the images of the three types of *A. lumbricoides* egg: (a) and (b) are infertile egg type that has not been fertilized, (c) and (d) are fertile egg type that has been mated, and (e) and (f) are decorticate egg type which is a parasitic egg similar to both types mentioned previously. For our study, the training dataset contains 200 images in each type; thus, total training images are 200 × 3 = 600.

### 2.5. Experiment

This experiment is divided into two phases: firstly, finding suitable number of convolution layer; secondly, the model from first step is optimized by perform parameter adjustment. The overall system is shown in Figure 3.

**Figure 5:**
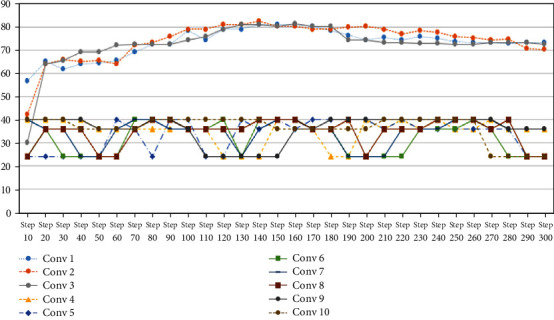
Comparison of the accuracy of the convolution layer without ReLu, maxpool, and dropout.

Figure 3 shows the experimental method of the research, divided into 2 phases and divided the images into 2 groups: training group and test group. The training data is sent to the first convolution layer of a CNN learns to recognize images with high accuracy. Then, increase the number of layers from 2 to 10 layers, which in this phase will not have stimulation, reduction, and dropout functions, so only convolution and fully connected layers will be performed (see Figure 5).

**Figure 6:**
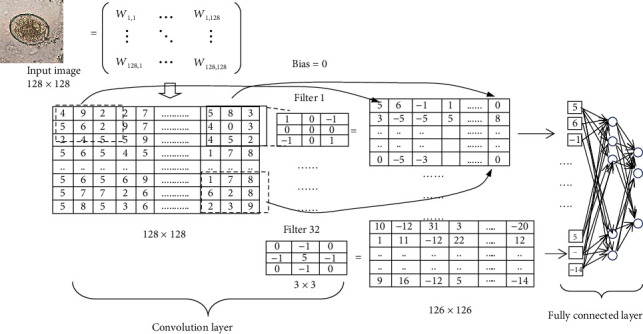
Example shape calculator for convolutional layers.

Figure 6 shows how to convolute the image with a filter which in this experiment has 32 filters, 3 × 3 kernel size, and bias = 0. Then, calculate according to equation (1), for example, five masking perform by (4 × 1) + (9 × 0) + (2 × (−1)) + (5 × 0) + (6 × 0) + (2 × 0) + (2 × (−1)) + (4 × 0) + (5 × 1) + 0. Phase 2 uses the first three highest accuracy layers to optimize the architecture with the addition of stimulus, reduction, and dropout functions, and the architecture adjustment is shown in Table 1, and the results are shown in the figure. The results of classification of test set images are shown in Table 2.

## 3. Results and Discussion

### 3.1. Results

After the training step, CNNs will create a model that produces results with long-time processing and low accuracy as shown in Figure 6; then, the authors' tuning model by adding conditions with maxpooling Figure 6 shows classification results in which the *x*-axis and *y*-axis represent learning step and classification accuracy, respectively. The results state that setting the convolution layer as one, two, and three is clearly outperform than the others. The accuracy is significantly dropped from four to ten layers (Excessive setting of the convolution layer causes misclassification problem. Consequently, dominant features lost its characteristic when transfer to other layers). Therefore, the top three accuracy settings are chosen in next phase. The next phase experiment is performed by adding the tuning steps. The additional steps consist of ReLu, maxpooling, and dropout, respectively. . Then, top three accuracy models from the first phase are fed into second phase to adjust ReLu activation maxpooling and dropout values, which can be rewritten as shown in Table 1. To measure the image classification accuracy, the researcher uses a confusion matrix and finds precision, recall, and accuracy, as shown in equations (3), (4), and (5).

(3) $$\begin{array}{c} \text{Precision}=\frac{\text{TP}}{\left(\text{TP}+\text{FP}\right)}, \end{array}$$

(4) $$\begin{array}{c} \text{Recall}=\frac{\text{TP}}{\left(\text{TP}+\text{FN}\right)}, \end{array}$$

(5) $$\begin{array}{c} \text{acc}=\frac{\text{TP}+\text{TN}}{\left(\text{TP}+\text{TN}+\text{FP}+\text{FN}\right)}, \end{array}$$

where true positive (TP) is predicting yes, and the answer is yes (Hit). True negative (TN) is predicting no, and the answer is really no (correct rejection). False positive (FP) is predicting yes, but the answer is no (false alarm). False negative (FN) is a prediction of no, but the answer is yes (Miss). acc is the number of times the prediction is divided by the total number of prediction. *Recall* is the completeness of the ratio. It is the ratio of correct prediction based on total number of valid data. *Precision* is the ratio of correct prediction based on the amount of data retrieved.

**Figure 7:**
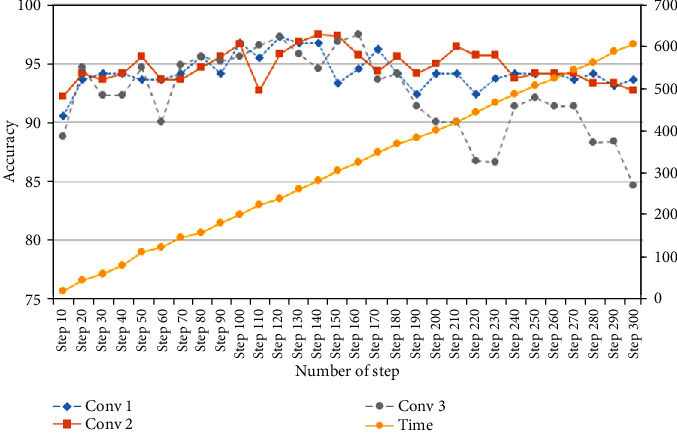
Result of accuracy and time of training with our CNN architecture.

Input() is a 3-channel 128 × 128 input image (R, G, B). Conv() is convolution. Re*Lu*() is the use of the activate function ReLu. *Maxpool*() is downsampling, and drop() is the reduce number of nodes. The tuning results are shown in Figure 7.

Figure 7 presented the time and accuracy of the tuning model. The step will notice that in each architecture, the accuracy results exceed more than 90% since the twentieth step.

In Table 2, the result shows visual prediction by choosing the unknown image of parasite eggs that enhances the number of 45 images.

### 3.2. Discussion

**Figure 8:**
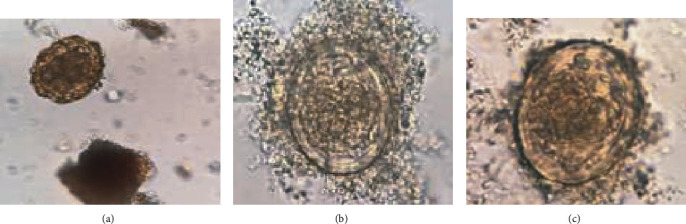
The 3 wrong predictions. (a) Fertile type, but predicted as infertile type. (b, c) Images of a decorticate type but predicted a fertilized.

In this experiment, the first phase performs straightforwardly to search for a suitable amount of convolution layer, and the results are shown in Figure 6. For the second phase, the top three accuracies of convolution layers are selected to perform further experiments. The second phase experiment performs by adding a fine-tuning step in the designed CNNs, and the experimental results are shown in Figure 7. The first convolution layer duty is capturing the low-level features such as edges or colour if sample image characteristic is obviously different, and the valuable features are clearly extracted in the first convolution. The authors trained CNNs with a few layers and then increase it slightly to obtain more accuracy until no more improvement. The reason is that some features of one image may become features of another. Suppose train a model for detecting infertile type if all features are detected and add more layers, and it can start detecting everything in the image that is considered to be part of the infertile type. Therefore, it may sometimes classify the image of another type with infertile (see Figure 8). Therefore, adding excessive layers causes the misclassification problem significantly. The features may lose its characteristic during transfer among layers.

Even though each image of datasets surrounds with artefacts, those artefacts did not increase uniqueness. The unique characteristics of *A. lumbricoides* egg (fertile and infertile eggs) under microscope are identified by the round to oval shapes, size (40 × 60 *μ*m for fertile egg and 40 × 90 *μ*m for infertile egg), and thick egg shell with typical chitinous layer (thick in fertile egg and quite thin for infertile egg), and the outer most layer revealed albuminous coat. So, the feature of dirt/artifacts has no chance to fit all of identified characters as described above.

## 4. Conclusions

In this experiment, the authors find the suitable number of convolution layers for all 3 parasitic eggs. First, this experiment stops at three layers because beyond there was no more improvement in the classify accuracy. The second phase is choosing three mentioned layers to perform a further experiment by tuning the CNNs with ReLu maxpool and dropout, respectively, to find the model that provides the highest accuracy. Focusing on the classification performance for classification parasite egg type, we choose the classification accuracy as objective evaluation criteria and compared it with outcomes mentioned in other papers which are also based on the CNN architecture. The developed model is useful for medical informatics, image recognition. The limitation of the research is the manual tuning of parameters. In future work, the researchers will eliminate the limitations by automating optimization of the further.

## Figures and Tables

**Table 1:** The proposed CNN architecture.

Number of layer	Architecture
1 layer	Input(128, 128, 3) → Conv(3, 32) → ReLu() → maxpool(2, 2) → drop(0.5) → full*y*()
2 layers	Input128,128,3→Conv3,32→ReLu→maxpool2,2→drop0.5→Conv3,10→ReLu→maxpool2,2→drop0.5→fully
3 layers	Input128,128,3→Conv3,32→ReLu→maxpool2,2→drop0.5→Conv3,10→ReLu→maxpool2,2→drop0.5→Conv3,10→ReLu→maxpool2,2→drop0.5→fully

**Table 2:** Classification result of parasite egg type.

	Infertile	Fertile	Decorticate	Precision (%)	acc (%)
Infertile	15	0	0	100	
Fertile	1	14	0	93.3	
Decorticate	0	2	13	86.6	
Recall (%)	93.7	87.5	92.8		
					93.33

## Data Availability

The datasets are belonging to parasite laboratory in Srinagarind Hospital which is located in Khon Kean University, Thailand. The datasets are confident due to the university policy, so we cannot share as a public.
